# Gastrodin and Microglial Autophagy–Pyroptosis Crosstalk: Evidence and Hypotheses Involving the AMPK/mTOR/NLRP3 Axis

**DOI:** 10.3390/ijms27198754

**Published:** 2026-09-30

**Authors:** Chan-Yen Kuo, I-Shiang Tzeng, Yi-Chyan Chen, Mao-Liang Chen

**Affiliations:** 1 Institute of Oral Medicine and Materials, College of Medicine, Tzu Chi University, Hualien 97004, Taiwan; 2 Department of Medical Research, Taipei Tzu Chi Hospital, Buddhist Tzu Chi Medical Foundation, New Taipei City 231016, Taiwan; 3 Department of Psychiatry, Taipei Tzu Chi Hospital, Buddhist Tzu Chi Medical Foundation, New Taipei City 231016, Taiwan

**Keywords:** gastrodin, microglia, autophagy, pyroptosis, NLRP3 inflammasome

## Abstract

Gastrodin (GAS), a bioactive glycoside from *Gastrodia elata*, shows anti-inflammatory and neuroprotective activity in preclinical models. This narrative review evaluates whether autophagy, mitochondrial quality control, and NOD-like receptor family pyrin domain containing 3 (NLRP3)-dependent pyroptosis may contribute to its effects on microglia. Studies in microglial cultures and animal models report reduced inflammatory activation and changes in microglial phenotype after GAS treatment. Related studies in endothelial cells, neurons, macrophages, and whole brain tissue associate GAS with AMP-activated protein kinase (AMPK) signaling, mechanistic target of rapamycin (mTOR) inhibition, autophagy or mitophagy markers, and lower inflammasome or pyroptosis markers. These findings support an AMPK/mTOR/NLRP3-centered working model, but the complete causal chain has not been demonstrated in microglia within a single experimental system. We distinguish microglia-specific observations from evidence extrapolated across cell types and disease models, compare GAS with its metabolite p-hydroxybenzyl alcohol, and discuss limitations of inhibitor-based experiments and variation in dose and sampling time. Inducible microglial genetic deletion, flux measurements, target-engagement assays, and matched pharmacokinetic studies are needed to test this model. Human evidence remains insufficient to confirm the proposed microglial mechanism or its clinical relevance.

## 1. Introduction

### 1.1. Microglial Homeostasis and the Onset of Neuroinflammation

Microglia are the primary innate immune sentinels of the central nervous system (CNS), maintaining parenchymal homeostasis, supporting synaptic remodeling, and preserving neuronal integrity [1]. Under physiological conditions, ramified surveillance microglia monitor the brain microenvironment and respond rapidly to perturbations in biochemical signaling, metabolism, and tissue mechanics [2]. Acute insults—such as ischemic stroke and traumatic brain injury (TBI) or chronic exposure to pathogen- and damage-associated molecular patterns (PAMPs and DAMPs)—induce morphological, transcriptional, and functional transitions in microglia [3,4]. This phenotypic polarization, historically classified along a continuum from pro-inflammatory cytotoxic to anti-inflammatory resolving states, constitutes an endogenous adaptive response [5,6].

Dysregulated microglial overactivation initiates an escalating neuroinflammatory cascade that worsens secondary tissue damage in both acute and chronic neurological conditions, including Alzheimer’s disease (AD), Parkinson’s disease (PD), and ischemic stroke [1,7,8]. Chronic microglial activation upregulates and releases pro-inflammatory mediators including inducible nitric oxide synthase (iNOS), cyclooxygenase-2 (COX-2), tumor necrosis factor-alpha (TNF-α), interleukin-6 (IL-6), and interleukin-1β (IL-1β) primarilythrough nuclear factor-κB (NF-κB) nuclear translocation and mitogen-activated protein kinase (MAPK) phosphorylation [4,9]. When this cytotoxic response overwhelms endogenous clearance mechanisms, accumulated reactive oxygen species (ROS) and reactive nitrogen species compromise blood–brain barrier (BBB) integrity, impair microvasculature, trigger neuronal apoptosis, and disrupt synaptic plasticity [10,11,12]. Consequently, suppressing excessive microglial activation, inhibiting pro-inflammatory polarization, and restoring homeostatic function remain central goals in treating neurodegenerative and neuroinflammatory disorders [2,13].

### 1.2. Autophagy–Pyroptosis Crosstalk in Microglial Regulation

Microglial phenotypic equilibrium and inflammatory quiescence depend on functional interplay between macroautophagy (autophagy) and pyroptosis [3,14]. Autophagy is a conserved lysosomal degradation pathway that clears protein aggregates, dysfunctional organelles, and intracellular pathogens, maintaining bioenergetics and organellar quality control [15,16]. In contrast, pyroptosis is a lytic, pro-inflammatory form of programmed cell death driven by the NOD-like receptor family pyrin domain containing 3 (NLRP3) inflammasome complex [16,17]. Cellular danger cues induce the NLRP3 sensor protein to recruit caspase activation and recruitment domain (CARD)-containing apoptosis-associated speck-like protein (ASC) and pro-caspase-1, triggering caspase-1 autoprocessing and activation [3,18]. Active caspase-1 then cleaves gasdermin D (GSDMD), releasing its N-terminal domain (GSDMD-N) to oligomerize and form plasma membrane pores, inducing osmotic lysis and releasing mature IL-1β and interleukin-18 (IL-18) [3,10].

Microglial autophagy represses NLRP3 inflammasome activation and pyroptosis [3,14]. Under basal conditions, selective mitochondrial autophagy (mitophagy), largely regulated by the PTEN-induced kinase 1 (PINK1)/Parkin E3 ubiquitin ligase pathway, degrades depolarized and damaged mitochondria [19,20]. Impaired autophagic flux leads to the accumulation of dysfunctional mitochondria, generating excess mitochondrial ROS (mtROS) and releasing oxidized mitochondrial DNA (ox-mtDNA) into the cytosol [14,19]. These mitochondrial alterations drive inflammasome activation: mtROS enhances NF-κB-dependent transcriptional upregulation of NLRP3 and pro-IL-1β (Signal 1: priming), whereas ox-mtDNA and downstream ion fluxes directly promote NLRP3 assembly and ASC speck formation (Signal 2: activation) [14,17,21]. Functional autophagic flux directs cytoplasmic NLRP3 complexes and ASC specks toward autolysosomal clearance, preventing GSDMD cleavage and membrane pore formation [3,10]. Restoring autophagic flux to suppress NLRP3–GSDMD-mediated pyroptosis thus preserves microglial viability and attenuates CNS neuroinflammation [3,14,17].

Recent reviews have synthesized how autophagy and mitochondrial quality control may constrain NLRP3-dependent neuroinflammation across neurological disorders, particularly Alzheimer’s and Parkinson’s diseases [22,23]. They provide a broader framework for interpreting autophagy–inflammasome interactions; however, these reviews do not establish the proposed sequence as a gastrodin-specific mechanism in microglia.

### 1.3. Gastrodin: Mechanisms and Multitarget Potential

Gastrodin (GAS; 4-hydroxybenzyl alcohol 4-O-β-D-glucopyranoside) is the major bioactive phenolic glycoside isolated from the rhizome of *Gastrodia elata* Blume [24,25]. Long prescribed in East Asian medicine for vertigo, headache, and motor dysfunction, GAS penetrates the BBB after systemic administration and confers neuroprotection [5,8,26]. Pharmacological evaluations show that GAS exerts anti-inflammatory, antioxidant, anti-apoptotic, and neurotrophic actions in models of cerebral ischemia–reperfusion injury (CIRI), TBI, neuropathic pain, epilepsy, and cognitive impairment [3,17,27,28,29]. In vivo pharmacokinetic analyses confirm that GAS is converted to its active aglycone, 4-hydroxybenzyl alcohol (HBA; gastrodigenin), which distributes broadly across CNS tissues [7,8,30].

At the molecular level, studies in several neural and non-neural systems associate GAS with AMP-activated protein kinase (AMPK) phosphorylation and changes in mechanistic target of rapamycin (mTOR), autophagy, and inflammatory signaling [7,15,31,32]. Protein arginine N-methyltransferase 1 (PRMT1)–AMPK modulation has been investigated in brain microvascular endothelial cells, whereas other autophagy and mitophagy branches come from distinct tissues and models [15,19,31,33]. Separate preclinical experiments report lower NF-κB, NLRP3, caspase-1, and GSDMD readouts following GAS treatment [3,10,17]. Together these observations motivate, but do not establish, a unified AMPK–autophagy–NLRP3 causal pathway in microglia.

Although evidence demonstrates that GAS suppresses microglial activation and supports functional recovery, its integrated metabolic, autophagic, and anti-pyroptotic mechanisms remain fragmented across diverse models. A comprehensive review of how GAS regulates the AMPK/mTOR/NLRP3 axis to control microglial autophagy–pyroptosis crosstalk has been lacking. This review synthesizes the pharmacological actions of GAS, covering its pharmacokinetic profile, BBB permeability, modulation of the AMPK/mTOR autophagic cascade and PINK1/Parkin-mediated mitophagy, and inhibition of the NLRP3/caspase-1/GSDMD pyroptotic pathway. Through this integrated framework, we outline how GAS restores microglial immunometabolism, evaluate its translational therapeutic potential in neuroinflammatory disease, and address primary clinical development barriers. Unlike accounts that consider autophagy and pyroptosis independently, this review traces a proposed sequence from AMPK/mTOR-dependent autophagic and mitochondrial quality control to reduced NLRP3 priming and activation, while distinguishing direct experimental observations from mechanistic links that still require causal testing. Throughout this review, findings obtained directly in microglia are distinguished from supporting observations in whole brain tissue, brain microvascular endothelial cells, neurons, and non-neural systems; the diagrams synthesize these data as hypotheses rather than single experimentally proven pathways.

## 2. Central Nervous System Bioavailability and Active Metabolites of Gastrodin

### 2.1. Blood–Brain Barrier Penetration Dynamics

**Figure 1:**
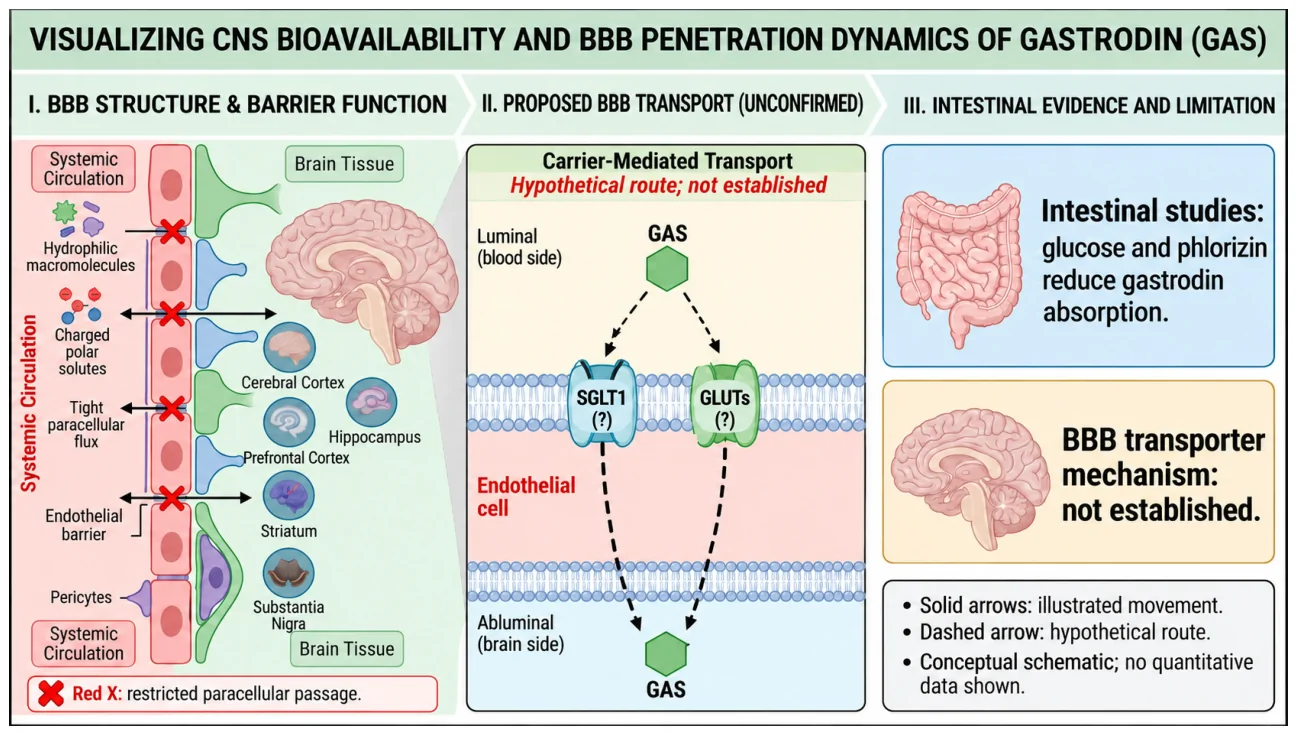
Central nervous system exposure and proposed blood–brain barrier transport of gastrodin. The diagram illustrates regional distribution and a hypothetical glucose-transporter route. Panel III adapts intestinal transport findings for gastrodin analogues from Guo et al. [35]; these findings do not establish SGLT1- or GLUT-mediated transport across the blood–brain barrier. CNS exposure is supported by pharmacokinetic studies [24,25], whereas the specific endothelial transport mechanism remains unconfirmed. (The original schematic was created with Gemini assistance, https://gemini.google.com/app?hl=en-IN, accessed on 10 September 2026. The final figure was revised using ChatGPT’s image-generation tool on 10 September 2026 and reviewed by the authors, https://chatgpt.com/). The authors reviewed and verified the final figure. Abbreviations: BBB, blood–brain barrier; CNS, central nervous system; GAS, gastrodin; GLUT, facilitative glucose transporter; SGLT1, sodium-glucose cotransporter 1.

The therapeutic success of CNS neuroprotective candidates depends on their ability to cross the BBB [8]. Brain capillary endothelial cells, sealed by tight junctions, pericytes, and astrocytic end-feet, restrict the paracellular and transcellular diffusion of hydrophilic macromolecules and charged solutes into the cerebral parenchyma [8,34]. Structurally, GAS contains an aglycone core conjugated to a D-glucopyranosyl moiety, resulting in high hydrophilicity and low lipid solubility [25,35]. Despite these properties, systemically administered GAS crosses the BBB and distributes to the cortex, hippocampus, striatum, and substantia nigra (Figure 1) [5,8,24].

Systemically administered GAS has been detected in CNS compartments, but its route across the BBB has not been established [8,24]. Experiments with gastrodin analogues implicate glucose transport pathways in intestinal absorption [35]. Those intestinal findings cannot demonstrate sodium-glucose cotransporter 1 (SGLT1)- or facilitative glucose transporter (GLUT)-mediated passage of GAS through brain endothelial cells. Dedicated BBB transport studies, including transporter-specific perturbation and matched plasma-to-brain exposure measurements, are needed to distinguish passive and carrier-mediated contributions.

Following systemic administration, GAS can be detected in cerebrospinal fluid (CSF) and brain extracellular fluid, but the extent of CNS exposure depends on dose, route, and sampling compartment (Table 1). In rats given 200 mg/kg intravenously, the reported CSF-to-plasma area under the concentration–time curve (AUC) ratio was 4.8 ± 2.4%, while regional brain-dialysate-to-plasma ratios were 3.0–6.1%; these values do not establish uptake by microglia. A study of a 200 mg oral GAS capsule in humans quantified plasma exposure but did not measure CSF or brain concentrations (Table 1). Accordingly, human CNS exposure and its relationship to microglial target engagement remain undetermined. In pathological conditions such as CIRI and septic encephalopathy, BBB disruption may alter local exposure, although matched GAS and HBA measurements under these conditions are limited [10,34,36,37,38]. Delivery platforms have been investigated to alter exposure, but their microglial targeting requires direct pharmacokinetic validation [8,21].

### 2.2. Pharmacological Roles of Gastrodin vs. P-Hydroxybenzyl Alcohol

Whether the in vivo neuroprotective actions of *G. elata* stem from intact parent GAS or its aglycone metabolite, p-hydroxybenzyl alcohol (HBA; gastrodigenin), remains debated [8,30,39]. Intestinal and tissue β-glucosidases can convert GAS to HBA [8,30]. HBA lacks the glucosyl moiety and differs from GAS in physicochemical properties [8,35]. HBA has been detected in rat brain dialysate after intravenous GAS administration, and oral GAS produces measurable plasma HBA (Table 1). These data do not establish uniformly high brain HBA concentrations or show that GAS acts primarily as a prodrug; paired unbound CNS exposure after clinically relevant dosing remains to be established.

Beyond hydrolysis, a rat-plasma liquid chromatography–tandem mass spectrometry (LC–MS/MS) study after GAS administration identified HBA, p-hydroxybenzaldehyde, p-hydroxybenzoic acid, and oxidized glucoside derivatives [40]. These findings indicate additional oxidative transformations but do not establish the responsible enzymes or the concentrations of these products in microglia. Glucuronidation, sulfation, and methylation are possible subsequent transformations of phenolic species; their quantitative contributions after purified GAS dosing and the activities of the resulting conjugates remain inadequately characterized. Accordingly, AMPK activation observed after GAS administration in vivo cannot yet be assigned specifically to unchanged GAS, HBA, or another metabolite. Time-resolved measurements of unbound parent compound and metabolites in plasma and brain, together with direct comparative AMPK assays and selective perturbation of metabolic pathways, are needed to determine the active species.

Pharmacodynamic analyses, however, demonstrate distinct and complementary activities rather than a simple prodrug relationship [8,39]. In two-vessel occlusion (2-VO) models of chronic cerebral hypoperfusion, equimolar doses of GAS and HBA (25 and 50 mg/kg) produced comparable reductions in cognitive deficits, shortened Morris water maze escape latencies, and attenuated hippocampal neuronal injury [39]. Quantitative metabolomics confirmed that both compounds restored glycolytic flux, tricarboxylic acid (TCA) cycle intermediates, and pentose phosphate pathway (PPP) activity, thereby preserving mitochondrial respiration in injured brain tissue [39]. Both agents also reduced parenchymal levels of amyloid-beta (Aβ1-42) and phosphorylated tau (*p*-tau396 and *p*-tau217) [8,39].

Spectrum-effect studies in lipopolysaccharide (LPS)-activated BV-2 microglia confirm functional divergence between the molecules [30]. While HBA functions primarily as a direct free-radical scavenger, parent GAS modulates intracellular inflammatory signaling [28,30]. Specifically, GAS binds regulatory kinases, inhibits IκB kinase (IKK) phosphorylation, and prevents NF-κB p65 nuclear translocation, thereby downregulating iNOS, COX-2, and inflammatory cytokine expression [4,9,30]. Cellular thermal shift assays (CETSA) and surface plasmon resonance (SPR) confirm physical binding between GAS and signaling kinases, including phosphoinositide 3-kinase (PI3K) [41]. Furthermore, parent GAS stabilizes vesicular monoamine transporter 2 (VMAT2) trafficking via mitogen-activated protein kinase kinase (MEK)-dependent pathways to preserve dopamine homeostasis [42]. Both agents protect dopaminergic neurons against rotenone and leucine-rich repeat kinase 2 (LRRK2) G2019S neurotoxicity [7,43].

GAS treatment has been associated with AMPK phosphorylation, altered autophagic signaling, and reduced inflammasome and pyroptosis markers in preclinical models [3,5,6,14,15,31]. GAS and HBA can each show neuroprotective effects [8,39], but comparisons of their intracellular signaling at matched unbound concentrations are limited. Because GAS is converted to HBA and additional metabolites, treatment-associated AMPK activation cannot be attributed to the parent compound solely from studies that administer GAS. The relative contributions of GAS, HBA, and other metabolites to microglial effects in vivo remain unresolved.

**Table 1:** Pharmacokinetic evidence relevant to GAS and HBA distribution in Section 2.1 and Section 2.2.

Primary Study and Model	Analyte; Administered Dose and Route	Systemic Pharmacokinetics	CNS Exposure or Interpretation
Ju et al., 2010; humans [44]	GAS; GAS capsule 200 mg orally	Plasma Cmax 1484.55 ± 285.05 ng/mL; Tmax 0.81 ± 0.16 h; AUC0–t 5619.41 ± 1972.88 ng·h/mL; AUC0–∞ 7210.26 ± 3472.74 ng·h/mL; t½β 6.06 ± 3.20 h.	CSF, brain/plasma AUC and regional distribution NR; human systemic data do not establish brain exposure.
Wang et al., 2007; rats [45]	GAS; 50 mg/kg intranasal or intravenous	Plasma numerical PK NR in available report summary.	Intranasal CSF AUC was comparable to intravenous CSF AUC; numerical CSF concentration and regional brain exposure NR.
Lin et al., 2007; rats [46]	GAS; 100 or 300 mg/kg intravenous	Unbound blood sampled by microdialysis; numerical Cmax/AUC NR in available summary.	Striatal extracellular GAS peaked approximately 15 min after injection; numerical brain/plasma AUC NR in available summary.
Wang et al., 2008; rats [47]	GAS; 200 mg/kg intravenous	Plasma AUC used as denominator; absolute plasma Cmax/AUC NR in available summary.	CSF/plasma AUC 4.8 ± 2.4%; frontal cortex 3.3 ± 1.2%; hippocampus 3.0 ± 0.7%; thalamus 3.3 ± 1.3%; cerebellum 6.1 ± 1.9%. HBA was low in CSF and plasma.
Lin et al., 2008; rats [48]	HBA formed after 50 mg/kg intravenous GAS	HBA blood Cmax, AUC and t½ NR in available summary.	HBA detected in brain dialysate and bile by 10 min and peaked around 15 min; this is metabolite exposure after GAS dosing, not direct HBA dosing.
Nepal et al., 2019; rats [49]	HBA formed after 40 mg/kg oral GAS	Plasma HBA: control Cmax 4.2 µg/mL and AUC 7.0 µg·h/mL; antibiotic-pretreated Cmax 2.5 µg/mL and AUC 4.6 µg·h/mL.	CSF and regional brain HBA NR; altered plasma HBA formation cannot establish brain concentration.
Huang et al., 2023; rats and mice [50]	GAS; oral (dose details NR in available summary)	Absolute oral bioavailability: Wistar rats 84.7%, Sprague–Dawley rats 60.6%, Kunming mice 70.8%, C57BL/6J mice 49.9%.	Strain-dependent systemic F does not establish BBB penetration or human oral F.

Abbreviations: AUC, area under the concentration–time curve; Cmax, peak concentration; CSF, cerebrospinal fluid; F, absolute bioavailability; GAS, gastrodin; HBA, p-hydroxybenzyl alcohol; NR, value not available in the cited study summary; Tmax, time to peak; t½β, terminal half-life. Values are not pooled across species, dose, route, and compartment. Plasma protein binding for GAS and HBA and human brain concentrations remain inadequately documented in the sources tabulated here.

## 3. Activating the Autophagic Engine: The AMP-Activated Protein Kinase and Mechanistic Target of Rapamycin Pathway

### 3.1. AMP-Activated Protein Kinase (AMPK) Phosphorylation as the Primary Energy Sensor

AMP-activated protein kinase (AMPK) is the master regulator of cellular energy homeostasis and metabolic stress responses [19,51]. As a heterotrimeric serine/threonine kinase with a catalytic α subunit and regulatory β and γ subunits, AMPK senses increases in the AMP/ATP and ADP/ATP ratios. These shifts trigger conformational changes that promote phosphorylation of Thr172 in the α subunit’s activation loop [15,31]. In CNS pathologies—such as ischemia–reperfusion injury, oxidative stress, LPS exposure, or nutrient depletion—microglia exhibit ATP depletion, mitochondrial depolarization, and reduced basal AMPK phosphorylation [14,19,31]. This metabolic deficit impairs catabolic clearance, driving pro-inflammatory polarization and cell death [14,39].

GAS-associated AMPK signaling has been reported across neural, vascular, and non-neural models [15,31,51]. In cerebral ischemia–reperfusion experiments involving brain microvascular endothelial cells, PRMT1 downregulation accompanies restoration of AMPKα Thr172 phosphorylation [31]. Dorsomorphin (Compound C) attenuates several GAS-associated effects in these systems, consistent with AMPK pathway involvement but insufficient on its own to prove AMPK necessity because of inhibitor off-target activity [31,51,52]. GAS-associated forkhead box O1 (FoxO1)/transcription factor EB (TFEB) changes were demonstrated in foam cells [15], and acetyl-CoA carboxylase (ACC) phosphorylation has been reported in other metabolic settings [32]; neither result establishes the same downstream sequence in microglia.

### 3.2. Mechanistic Target of Rapamycin Complex 1 Inhibition and Initiation of Autophagic Flux

The mechanistic target of rapamycin complex 1 (mTORC1) couples AMPK phosphorylation to macroautophagy initiation [33]. As an anabolic regulator, mTORC1 coordinates nutrient availability with protein synthesis and suppresses autophagy initiation [16,33]. Under basal conditions, active mTORC1 phosphorylates Unc-51-like autophagy-activating kinase 1 (ULK1) at Ser757, dissociating the ULK1-AMPK complex and inactivating the autophagic machinery [15,33].

GAS regulates this molecular switch via AMPK [14,33]. Activated AMPK inhibits mTORC1 by directly phosphorylating its Raptor subunit and by activating the upstream tuberous sclerosis complex 2 (TSC2) [33]. Consequently, GAS reduces phosphorylation of mTOR (p-mTOR/mTOR) and its substrate, the eukaryotic translation initiation factor 4E-binding protein 1 (p-4EBP1/4EBP1), thereby relieving repression of autophagic induction [33]. With mTORC1 inhibition relieved, the ULK1 core complex phosphorylates class III phosphatidylinositol 3-kinase (PI3KC3/Vps34) and Beclin-1, initiating phagophore nucleation and recruiting downstream autophagy-related (Atg) conjugates [33,53].

In several neural and non-neural injury models, GAS changes Beclin-1, microtubule-associated protein 1 light chain 3-II (LC3-II), and sequestosome 1 (p62) readouts [33,53,54]. These steady-state markers cannot by themselves establish increased autophagic flux; dynamic lysosomal-blockade experiments are required [55]. Experiments with 3-methyladenine (3-MA) and bafilomycin A1 support an autophagy-related contribution in the models studied [33,54], but their effects and off-target limitations do not establish that autophagic flux is indispensable for GAS responses in microglia [55,56].

### 3.3. Evidence for Mitochondrial Quality Control Across Distinct Cell Types

Mitochondrial quality control influences inflammatory responses in microglia, but evidence that GAS restores microglial mitophagic flux remains limited [8,19,20]. Observations of mitochondrial membrane potential, reactive oxygen species, or mitochondrial morphology in whole brain tissue and non-microglial cells cannot establish microglia-specific mitophagy. The experimental system and measured endpoint must therefore be considered before extending a mitochondrial mechanism to microglia.

The PINK1/Parkin pathway provides a plausible route for removing damaged mitochondria. However, the GAS study cited for PINK1/Parkin-dependent mitophagy examined myocardial ischemia/reperfusion and cardiomyocytes, not microglia [20]. Its biochemical and ultrastructural findings support GAS-associated mitophagy in that cardiac setting. They do not demonstrate PINK1 stabilization, Parkin recruitment, or completed mitophagic flux in GAS-treated microglia. Direct microglial studies should combine mitochondrial-targeted flux reporters with PINK1 or Parkin loss-of-function and rescue experiments before assigning this pathway a causal role in the proposed autophagy–pyroptosis crosstalk.

**Figure 2:**
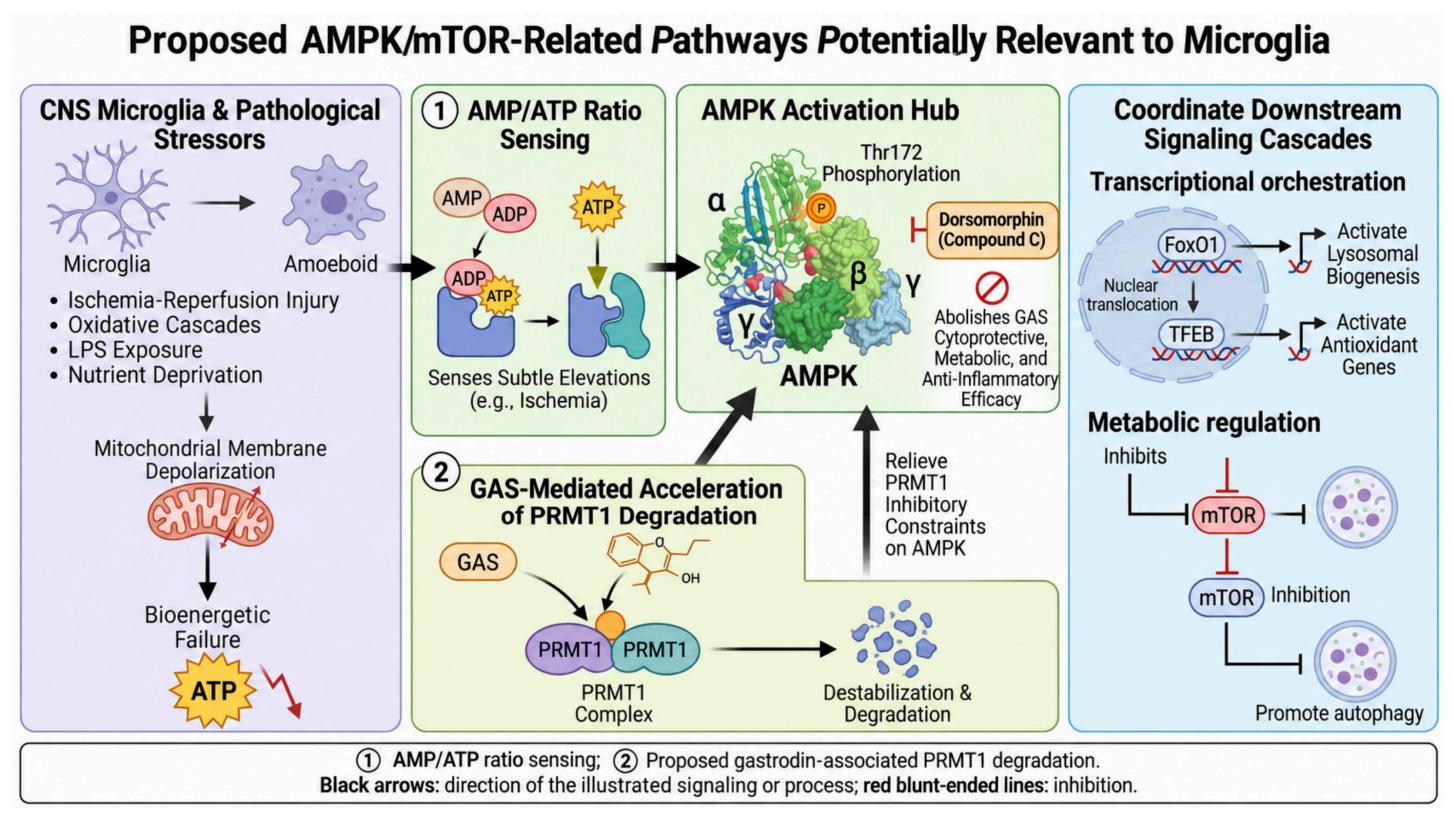
Proposed AMPK/mTOR-related pathways potentially relevant to microglia. The diagram integrates findings from distinct experimental systems and does not depict a demonstrated signaling cascade in microglia. ① indicates AMP/ATP ratio sensing; ② indicates the proposed gastrodin-associated degradation of PRMT1. Black arrows show the illustrated direction of signaling or a process, and blunt-ended lines indicate inhibition. Evidence for individual pathways includes PRMT1–AMPK signaling in cerebral ischemia [31] and AMPK-FoxO1-TFEB signaling in foam cells [15]; their combination in microglia remains a proposed framework. The original schematic was created with Gemini assistance and subsequently revised using ChatGPT’s image-generation tool. The Gemini model version was not recorded. Abbreviations: AMPK, AMP-activated protein kinase; CNS, central nervous system; FoxO1, forkhead box O1; GAS, gastrodin; LPS, lipopolysaccharide; mTOR, mechanistic target of rapamycin; PRMT1, protein arginine N-methyltransferase 1; TFEB, transcription factor EB.

The AMPK–optic atrophy 1 (OPA1) findings likewise come from middle cerebral artery occlusion/reperfusion rats and oxygen-glucose deprivation/reperfusion experiments in SH-SY5Y cells, a neuron-like line [19]. In these systems, GAS-associated AMPK activation and OPA1 expression accompanied mitochondrial fusion and improved bioenergetic readouts; AMPK silencing weakened several effects [19]. These results provide evidence for a neuronal or mixed-tissue mitochondrial response, but do not show that AMPK–OPA1 operates in microglia. Reports of sirtuin 3 (SIRT3)–mitochondrial transcription factor A (TFAM) signaling in vascular dementia and activated glial models represent additional, distinct experimental contexts [26,57]. The PINK1/Parkin, AMPK–OPA1, and SIRT3–TFAM branches are therefore presented as candidate contributors rather than a single GAS-induced sequence established in microglia (Figure 2).

## 4. Mitophagy-Driven Suppression of the NOD-like Receptor Family Pyrin Domain Containing 3 Inflammasome

### 4.1. Quenching Mitochondrial Reactive Oxygen Species to Inhibit Inflammasome Priming (Signal 1)

NLRP3 inflammasome activation requires transcriptional priming (Signal 1) followed by oligomerization and complex assembly (Signal 2) [10,18]. Priming begins when pattern-recognition receptor (PRR) ligands, such as LPS, TNF-α, or DAMPs, engage Toll-like receptor 4 (TLR4) or dedicated cytokine receptors on microglial membranes [4,11]. Downstream signaling through myeloid differentiation primary response 88 (MyD88) and tumor necrosis factor receptor-associated factor 6 (TRAF6) activates the IKK complex [4,30]. Phosphorylation and proteasomal degradation of IκBα release NF-κB p65/p50 heterodimers, enabling their nuclear translocation and inducing de novo transcription of NLRP3 and pro-IL-1β [4,14,18]. Because basal NLRP3 levels are insufficient to support complex assembly under homeostatic conditions, this transcriptional induction is the rate-limiting step for inflammasome activation [10,11].

Mitochondrial ROS generated by damaged electron transport chains amplify NF-κB-dependent priming [14,19]. In ischemic, traumatic, or metabolic stress microenvironments, loss of mitochondrial membrane potential induces electron leakage from complexes I and III, producing superoxide and hydrogen peroxide [19,58]. Excess mtROS oxidizes and inactivates regulatory phosphatases, prolonging IKK activity and sustaining NF-κB nuclear occupancy, thereby accelerating transcription of *NLRP3*, pro-IL-1β, and pro-IL-18 [10,14,18].

GAS suppresses this priming cascade by linking energy sensing to mitophagic clearance [14,19,20]. By stimulating AMPK phosphorylation and activating PINK1/Parkin-mediated mitophagy, GAS clears depolarized, ROS-generating mitochondria via autolysosomes [19,20,31]. This organellar clearance depletes the mtROS pool required to sustain NF-κB signaling [19]. Simultaneously, GAS suppresses the TLR4/TRAF6/NF-κB axis and activates peroxisome proliferator-activated receptor gamma (PPAR-γ), thereby reducing p65 phosphorylation and nuclear accumulation in activated microglia [4,18]. By eliminating mitochondrial sources of oxidative stress while downregulating inflammatory transcription factors, GAS suppresses Signal 1 priming [10,11,14].

### 4.2. Preventing Oxidized Mitochondrial DNA Releaseand NLRP3 Assembly (Signal 2)

Following transcriptional priming, macromolecular NLRP3 assembly requires a secondary activation stimulus (Signal 2) [10,17]. Signal 2 is triggered by cellular stressors, including purinergic receptor P2X7 (P2X7R) stimulation, potassium (K^+^) efflux, calcium (Ca^2+^) mobilization, and lysosomal rupture [3,59]. The release of damaged mitochondrial components specifically oxidized mitochondrial DNA (ox-mtDNA) and externalized cardiolipin directly nucleates the cytoplasmic NLRP3 complex [14,19,20]. Under neuroinflammatory stress, opening of the mitochondrial permeability transition pore (mPTP) releases fragmented ox-mtDNA into the cytosol, where it binds the NACHT domain of NLRP3 to induce an open conformation [14,26]. Oligomerized NLRP3 uses its pyrin domain (PYD) to recruit ASC through homotypic PYD–PYD interactions [59,60]. ASC aggregates into macromolecular specks that recruit pro-caspase-1 via caspase activation and recruitment domains (CARD-CARD interactions), assembling the functional inflammasome [10,18,60].

GAS prevents Signal 2 activation by preserving mitochondrial membrane integrity and blocking pro-inflammatory mitochondrial leakage [19,39]. Through AMPK–OPA1 activation, GAS stabilizes the inner mitochondrial membrane, preserves cristae architecture, and maintains mitochondrial membrane potential, thereby preventing mPTP opening and cytosolic ox-mtDNA release [19,26]. In parallel, GAS enhances SIRT3 activity, preserving TFAM-mediated mitochondrial DNA compaction and preventing oxidative matrix fragmentation [26,57]. In oxygen-glucose deprivation (OGD) and injury models, GAS inhibits perinuclear ASC speck formation, disrupts NLRP3-ASC binding, and suppresses downstream pro-caspase-1 recruitment [17,59,60].

**Figure 3:**
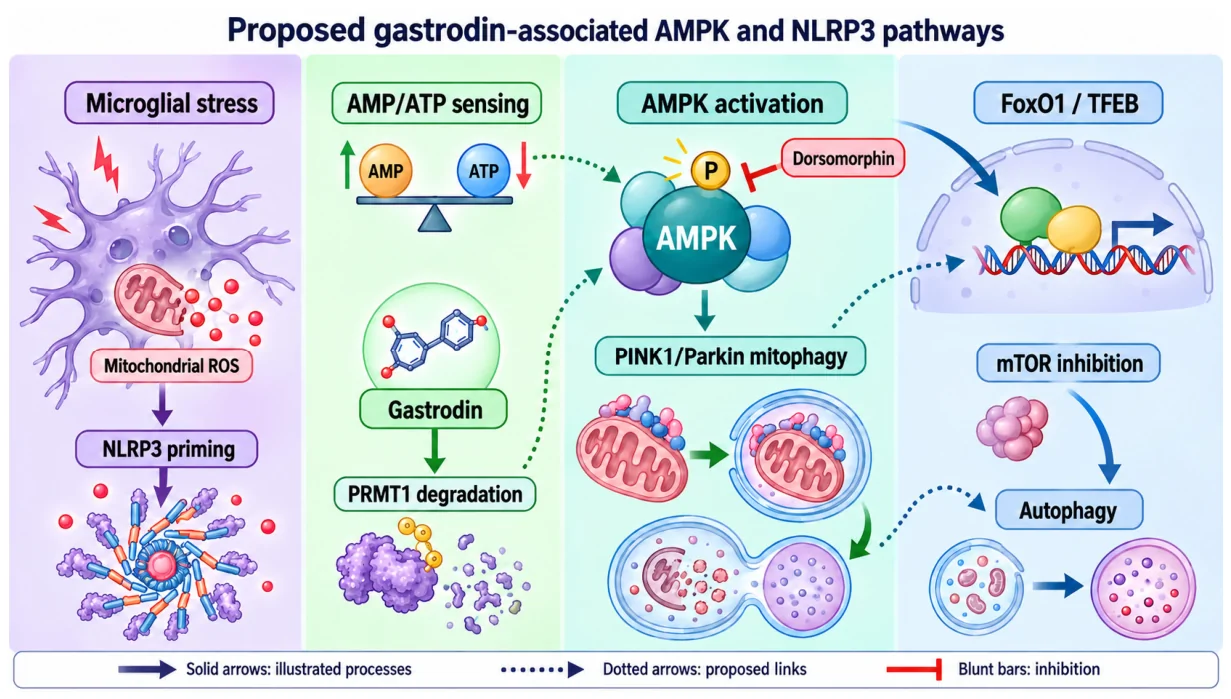
Proposed links between gastrodin-associated AMPK signaling, mitochondrial quality control, and NLRP3 inflammasome regulation. The schematic integrates findings from distinct experimental systems [10,17,19,31]. The complete pathway, including whether gastrodin-induced mitophagy reduces NLRP3 priming or assembly in microglia, has not been causally established. Solid arrows indicate illustrated processes, dotted arrows indicate proposed links across studies, and blunt-ended bars indicate inhibition. The original schematic was generated with Gemini assistance and subsequently revised using ChatGPT’s image-generation tool. The Gemini model version was not recorded. Abbreviations: AMP, adenosine monophosphate; AMPK, AMP-activated protein kinase; ATP, adenosine triphosphate; FoxO1, forkhead box O1; NLRP3, NOD-like receptor family pyrin domain containing 3; PINK1, PTEN-induced putative kinase 1; PRMT1, protein arginine N-methyltransferase 1; ROS, reactive oxygen species; TFEB, transcription factor EB; mTOR, mechanistic target of rapamycin.

GAS-induced macroautophagy also eliminates assembled inflammasome components [3,14]. Autophagosomes recognize polyubiquitinated ASC specks and assembled NLRP3 complexes via p62 adaptors and deliver them to lysosomes for degradation [15,54]. Inhibiting autophagic flux with 3-MA abolishes GAS’s ability to disrupt NLRP3 complexes, prevent ASC oligomerization, and attenuate inflammatory signaling [14,33]. Thus, GAS functions as a dual inhibitor of the NLRP3 pathway: it accelerates mitophagy to deplete mtROS (blocking Signal 1 priming) and preserves mitochondrial integrity to prevent cytosolic ox-mtDNA leakage and ASC speck assembly (blocking Signal 2 activation), thereby halting inflammasome execution before pyroptotic lysis begins (Figure 3) [3,10,14,19].

## 5. Halting the Pyroptotic Cascade

### 5.1. Inhibition of Caspase-1 Cleavage and Activation

Pyroptosis is a lytic, pro-inflammatory form of regulated cell death, distinguished from apoptosis by cellular swelling, early membrane rupture, and the release of intracellular contents [3,10]. In microglia and neural parenchyma exposed to CIRI, trauma, or sterile inflammation, canonical pyroptosis proceeds via caspase-1 activation [3,17]. Following NLRP3-ASC complex assembly, pro-caspase-1 binds ASC specks via homotypic CARD–CARD interactions [18,60]. This recruitment induces proximity-driven dimerization and autocatalytic cleavage of the 45-kDa pro-caspase-1 zymogen into active p20 and p10 subunits [17,18]. Active caspase-1 then cleaves pore-forming effectors and pro-inflammatory cytokine precursors [3,10].

GAS suppresses caspase-1 processing across multiple neurological and microvascular injury models [3,10,17]. In middle cerebral artery occlusion (MCAO) and in vitro oxygen–glucose deprivation/reperfusion (OGD/R) paradigms, ischemic insults trigger cleaved caspase-1 formation; GAS administration reduces p20 and p10 subunit levels without impairing basal cell viability [17,18]. Mechanistically, GAS suppresses upstream signal transducer and activator of transcription 3 (STAT3) and NF-κB signaling and disrupts long non-coding RNA nuclear paraspeckle assembly transcript 1 (lncRNA NEAT1)/microRNA-22-3p (miR-22-3p) signaling, thereby repressing NLRP3 transcription and oligomerization required for pro-caspase-1 auto-activation [17,59]. Furthermore, in TBI and spinal nerve ligation (SNL) models, GAS inhibits neural caspase-1 activation in a dose-dependent manner, achieving anti-inflammatory suppression comparable to selective inhibitors such as MCC950 [3,60]. By arresting caspase-1 activation at its upstream assembly node, GAS halts this proteolytic pathway before cellular destruction [3,10].

### 5.2. Blocking Gasdermin D-Mediated Pore Formation

The commitment step of pyroptosis is the formation of nanoscale plasma membrane pores by gasdermin D (GSDMD) [3,10]. Basally, full-length GSDMD adopts an autoinhibited conformation where the C-terminal domain (GSDMD-C) masks the cytotoxic N-terminal domain (GSDMD-N) [3,41]. Upon inflammasome assembly, caspase-1 cleaves a conserved aspartate site in the interdomain linker, releasing active GSDMD-N [10,41]. Liberated GSDMD-N translocates to the inner plasma membrane leaflet, binding phosphoinositides and cardiolipin [3,61]. Through oligomerization, 24 to 33 GSDMD-N protomers form transmembrane pores 10 to 18 nm in diameter [3,10]. These pores collapse transmembrane electrochemical gradients, driving water influx, cell swelling, and osmotic lysis characterized by lactate dehydrogenase (LDH) release [10,17].

GAS prevents this pore-forming cascade [3,10]. In endothelial and neural cultures exposed to hypoxia-reoxygenation or shear stress, GAS inhibits GSDMD-N cleavage [3,10]. By blocking caspase-1 activation and limiting GSDMD-N generation, GAS preserves membrane integrity, reduces extracellular LDH leakage, and inhibits propidium iodide/calcein influx [3,10,17]. In ischemic myocardium and brain parenchyma, GAS downregulates pro-pyroptotic signaling to preserve tissue architecture and peri-infarct borders [10,61]. Furthermore, by stimulating PI3K/Akt and nuclear factor erythroid 2-related factor 2 (Nrf2) antioxidant signaling, GAS limits lipid peroxidation, thereby preventing GSDMD-N insertion and oligomerization within membranes [8,41]. Consequently, GAS suppresses lytic membrane breakdown, shielding microglia and adjacent neural tissue from secondary necroinflammatory damage [3,60].

### 5.3. Suppressing the Release of Pro-Inflammatory Cytokines (Interleukin-1β and Interleukin-18)

GSDMD pore formation and cell lysis release mature cytokines that amplify neuroinflammation [10,17]. IL-1β and IL-18 are synthesized as inactive precursors (pro-IL-1β and pro-IL-18) lacking secretory signal peptides [18,59]. Caspase-1 proteolytically cleaves these precursors into mature forms, which exit via GSDMD-N pores or membrane rupture [3,10,17]. In the CNS parenchyma, extracellular IL-1β binds interleukin-1 receptor type 1 (IL-1R1) on astrocytes, endothelial cells, and neurons, inducing secondary production of IL-6, TNF-α, and matrix metalloproteinases (MMPs) that degrade tight junctions and disrupt the BBB [2,34]. Concurrently, mature IL-18 binds the IL-18 receptor complex to upregulate interferon-gamma (IFN-γ) and drive cytotoxic microglial polarization [5,59].

GAS inhibits the maturation and release of IL-1β and IL-18 [10,17,62]. In models of focal cerebral ischemia, septic shock, and neurogenic hypertension, GAS reduces mature IL-1β and IL-18 concentrations in brain tissue and plasma [17,18,62]. This reduction occurs via multi-tier inhibition: suppressing NF-κB p65 nuclear translocation decreases pro-IL-1β and pro-IL-18 transcription [4,18]; inhibiting NLRP3 assembly and caspase-1 activation prevents proteolytic cytokine maturation [10,17,62]; and blocking GSDMD pores restricts cytokine efflux across the membrane [3,10].

**Figure 4:**
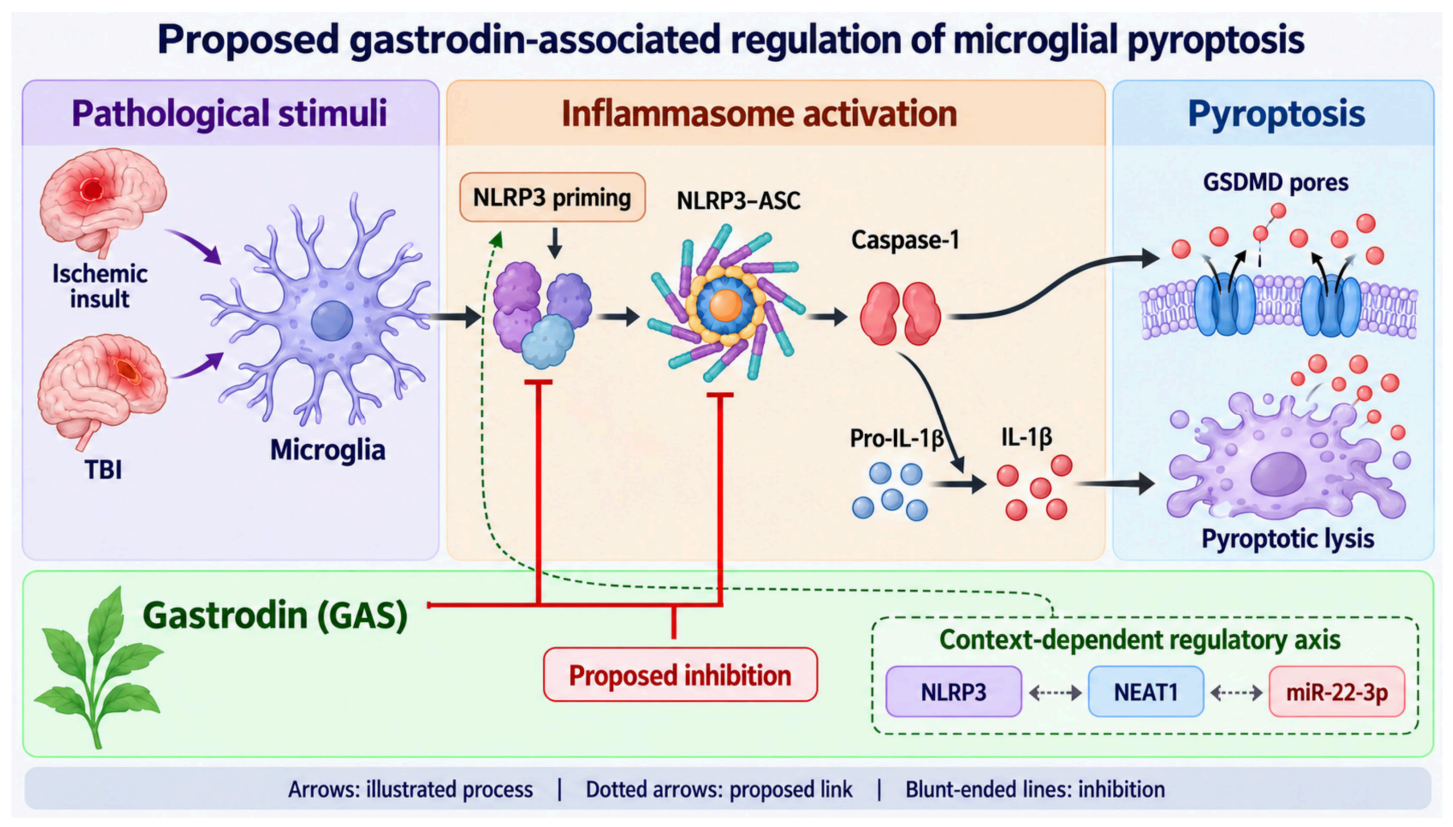
Proposed gastrodin-associated regulation of pyroptosis in preclinical injury models. The schematic summarizes findings from traumatic brain injury [3], cerebral ischemia [17], and microvascular reperfusion injury [10]. Changes in inflammasome and pyroptosis markers across these models do not establish the complete causal sequence in microglia. The NLRP3-NEAT1-miR-22-3p axis is shown as a context-dependent proposed link. Solid arrows indicate illustrated processes, dotted arrows indicate proposed links, and blunt-ended lines indicate proposed inhibition by gastrodin. The original schematic was generated with Gemini assistance and subsequently revised using ChatGPT’s image-generation tool; the Gemini model version was not recorded. Abbreviations: ASC, apoptosis-associated speck-like protein containing a CARD; CARD, caspase activation and recruitment domain; GAS, gastrodin; GSDMD, gasdermin D; IL-1β, interleukin-1 beta; miR-22-3p, microRNA-22-3p; NEAT1, nuclear paraspeckle assembly transcript 1; NLRP3, NOD-like receptor family pyrin domain containing 3; TBI, traumatic brain injury.

In triple-transgenic Alzheimer’s disease (3xTg-AD), TBI, and SNL models, GAS-mediated reductions in IL-1β and IL-18 correlate with decreased cerebral infarction, attenuated tau phosphorylation, reduced mechanical allodynia, and preserved hippocampal synaptic plasticity [3,17,34,60]. Through these actions, GAS interrupts the microglial pyroptotic cycle, attenuating neurovascular injury and supporting functional recovery (Figure 4) [3,10,17,18].

## 6. Remodeling Microglial Phenotypes: The Ultimate Cellular Outcome

### 6.1. Immunometabolic Reprogramming (From Glycolysis to Oxidative Phosphorylation)

Microglial functional plasticity depends on immunometabolic reprogramming [39,63]. Surveillance microglia primarily rely on mitochondrial oxidative phosphorylation (OXPHOS) and fatty acid oxidation (FAO) to support homeostatic functions [1,19]. Inflammatory or ischemic insults such as LPS, hypoxia-reoxygenation, or neurotoxins trigger a metabolic shift from mitochondrial respiration to aerobic glycolysis [39,63]. This shift upregulates hexokinase-2 (HK2), pyruvate kinase M2 (PKM2), and lactate dehydrogenase A (LDHA), accelerating glucose consumption, lactate accumulation, extracellular acidification, and cristae disruption [63,64]. Although this metabolic adaptation rapidly supplies ATP and biosynthetic intermediates for cytokine synthesis, it uncouples mitochondria, increases ROS production, and reduces oxidative phosphorylation capacity [39,63].

GAS reverses this glycolytic shift, restoring mitochondrial respiration and OXPHOS [19,39,63]. In models of metabolic neurotoxicity, GAS downregulates HK2, PKM2, and LDHA expression, lowering intracellular lactate and normalizing pyruvate levels [63]. This effect mirrors that of the HK2 inhibitor 2-deoxy-D-glucose (2-DG) and acts additively with it [63]. Metabolomic profiling shows that GAS and its metabolite HBA restore central carbon metabolism by replenishing TCA cycle intermediates and preserving PPP flux [39].

This metabolic restoration requires GAS-mediated activation of AMPK and SIRT3 [14,19,26]. Through the AMPK–OPA1 axis, GAS promotes inner mitochondrial membrane fusion, preserves cristae architecture, increases ATP synthesis, and normalizes the NAD^+^/NADH ratio [19]. At the same time, GAS maintains SIRT3-dependent deacetylation of TFAM, thereby preserving electron transport chain subunits and increasing the oxygen consumption rate (OCR) [26,57]. Mitophagy clears uncoupled mitochondria, ensuring that functional organelles sustain respiration [19,20]. By attenuating excessive glycolysis and restoring OXPHOS, GAS restricts the metabolic substrates required for pro-inflammatory gene expression, establishing an immunometabolic state that promotes microglial resolution [39,63].

### 6.2. Dampening M1-like Neurotoxicity and Promoting M2-like Neuroprotection

Microglial immunometabolism and autophagic clearance influence phenotypic states [5,6]. M1-like and M2-like labels describe selected activation features rather than fixed, mutually exclusive populations. Pro-inflammatory readouts include CD86, CD16/32, iNOS, nitric oxide (NO), TNF-α, IL-1β, and IL-6, whereas arginase 1 (Arg-1), chitinase-like 3 (Ym1/Chil3), and CD206/Mrc1 are commonly used as alternative-activation markers [4,5,6,9]. The presence of Arg-1 or CD206 does not by itself demonstrate secretion of IL-10, transforming growth factor-β (TGF-β), or neurotrophic factors, effective debris clearance, or neuronal repair.

In vitro and in vivo studies indicate that GAS shifts microglial polarization away from M1 activation toward an Arg-1^+^ M2-like phenotype [4,5,6,13]. In chronic systemic LPS-induced neuroinflammation, hippocampal microglia exhibit somal hypertrophy, process retraction, and cytokine secretion, which drive depressive- and anxiety-like behaviors [5]. GAS reverses these morphological alterations, restoring ramified morphology and inducing Arg-1 expression [5]. This phenotypic switch requires Nrf2 signaling; pharmacological Nrf2 inhibition with ML385 abolishes GAS-induced Arg-1 upregulation and its behavioral efficacy [5].

In spinal cord injury (SCI) models, GAS increased Arg-1 and CD206 and reduced iNOS and CD86 readouts, alongside improved locomotor and neuronal outcomes [6]. IL-10 and TGF-β were assessed in spinal cord homogenates, but these tissue-level measurements cannot establish microglia as their source [6]. Conditioned-medium experiments support altered glia–neuron communication, although they do not identify the relevant secreted mediator or establish that Arg-1 expression causes neuronal protection [6]. In an Alzheimer disease-related LPS model, GAS increased M2-associated markers while inhibiting measured microglial migration and phagocytosis [4]; thus, an Arg-1-positive shift should not be equated with greater clearance of pathogenic substrates. The PI3K/Akt inhibitor LY294002 diminished GAS-associated effects in the SCI study [6], but cell-specific causal links between signaling, secretion, and clearance require further testing.

Evidence for communication with astrocytes is emerging: in a BV-2 microglia-conditioned-medium/TNC-1 astrocyte system, GAS treatment was associated with higher astrocytic brain-derived neurotrophic factor (BDNF) and insulin-like growth factor 1 (IGF-1) expression [65]. This does not establish microglial secretion of BDNF or IGF-1, and glial cell line-derived neurotrophic factor (GDNF) release has not been demonstrated in the cited GAS microglial studies. The effects of GAS-associated Arg-1-positive states on uptake or degradation of Aβ, α-synuclein, myelin debris, apoptotic cells, and synapses have not been resolved with substrate-specific assays. Nor has a causal role been established for microglial proteolytic enzyme secretion or communication through fractalkine (CX3CL1)/its receptor CX3CR1, triggering receptor expressed on myeloid cells 2 (TREM2), complement, or exosomes. These are testable possibilities rather than demonstrated downstream functions.

**Figure 5:**
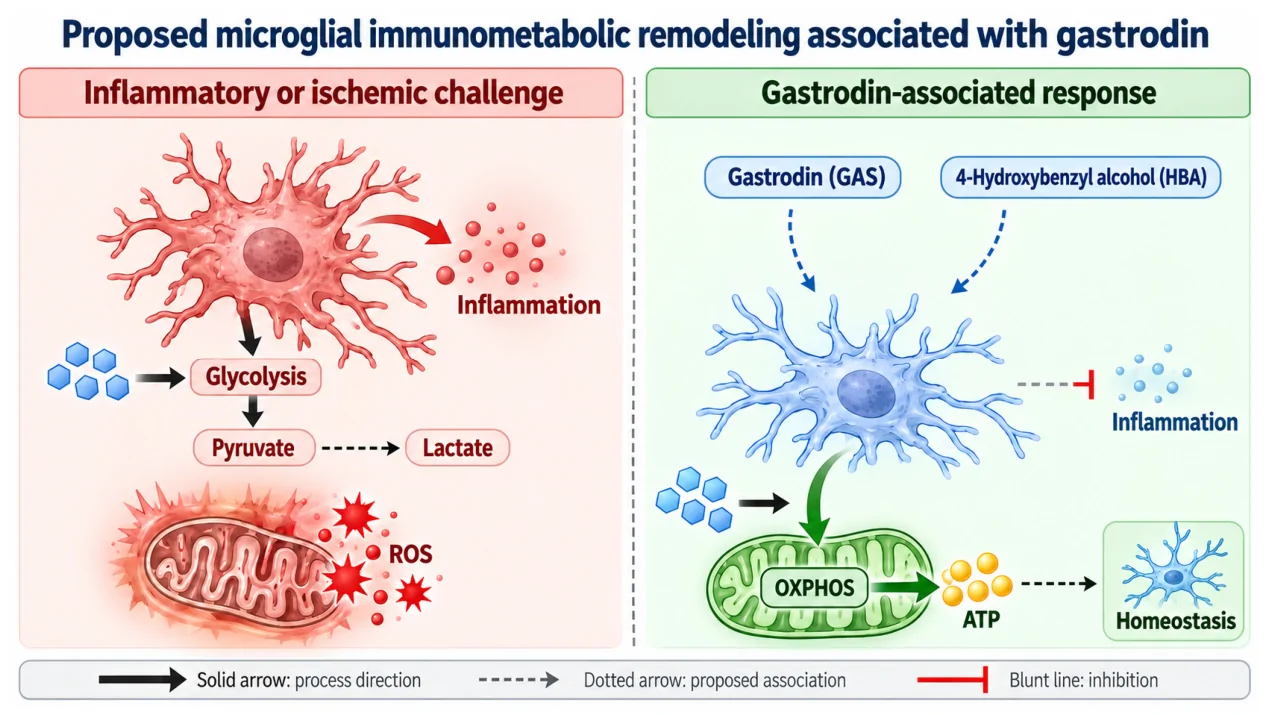
Proposed relationship between gastrodin-associated metabolic changes and microglial responses. The schematic contrasts inflammatory or ischemic challenge with a proposed response associated with gastrodin (GAS) or its metabolite p-hydroxybenzyl alcohol (HBA). Supporting evidence includes Nrf2-dependent, Arg-1-positive microglial responses in LPS-treated mice [5] and whole-brain energy-metabolism measurements in vascular dementia [39]. These findings come from different studies; the complete metabolic and phenotypic sequence depicted here has not been measured together in microglia. Solid arrows show the direction of illustrated processes, dotted arrows show proposed associations, and blunt-ended lines show inhibition. The vertical dashed line separates the two schematic conditions. The original schematic was prepared with Gemini (Google) assistance; this revised figure was redrawn with ChatGPT assistance. Abbreviations: ATP, adenosine triphosphate; GAS, gastrodin; HBA, p-hydroxybenzyl alcohol; OXPHOS, oxidative phosphorylation; ROS, reactive oxygen species.

Taken together, the current evidence supports GAS-associated changes in inflammatory markers, Arg-1/CD206 expression, and selected behavioral or tissue outcomes [4,5,6]. It does not yet establish that reduced pyroptosis or a metabolic shift causes a specific reparative microglial program, nor that such a program preserves the blood–brain barrier, prevents synaptic loss, or promotes debris clearance. Cell-resolved secretion measurements, defined phagocytic substrates, and co-cultures with neurons, astrocytes, oligodendrocytes, and endothelial cells are needed to test these proposed functions (Figure 5).

## 7. Pathological Relevance: The AMPK/mTOR/NLRP3 Axis in CNS Disorders

### 7.1. Cerebral Ischemia–Reperfusion Injury

CIRI involves bioenergetic failure, oxidative stress, microvascular injury, and secondary neuroinflammation [17,19]. Transient arterial occlusion followed by reperfusion introduces molecular oxygen into ischemic brain tissue, causing electron leakage from disrupted respiratory chains, a burst of ROS, and intracellular ATP depletion [19,31]. This energetic deficit suppresses AMPK signaling and triggers inflammatory cascades in microglia and brain microvascular endothelial cells (BMECs) [19,31]. Concurrently, ischemic stress upregulates long non-coding RNA (lncRNA) nuclear paraspeckle assembly transcript 1 (NEAT1), downregulates miR-22-3p, and activates STAT3/NF-κB cascades, driving NLRP3 transcription and assembly [17,59]. Active caspase-1 then cleaves GSDMD, triggering microglial and endothelial pyroptosis, which causes mature IL-1β and IL-18 release, disrupts BBB integrity, and expands cerebral infarction [10,17,36].

In ischemia/reperfusion experiments involving brain microvascular endothelial cells, GAS-associated PRMT1 reduction and AMPKα Thr172 phosphorylation accompanied protection against cellular injury [31]. Dorsomorphin weakened these effects, supporting pathway involvement in endothelial cells but not proving microglial AMPK dependence [31,52].

Downstream, GAS activates the AMPK-OPA1 cascade, promoting inner mitochondrial membrane fusion, restoring mitochondrial potential, elevating ATP production, and normalizing the NAD^+^/NADH ratio [19]. Concurrently, GAS counteracts ischemia-induced NEAT1/miR-22-3p and STAT3 activation to suppress downstream NLRP3-driven pyroptosis, thereby preserving BBB integrity, reducing infarct volume, and improving neurological scores [17,19,31,59].

### 7.2. Alzheimer’s and Parkinson’s Diseases

Alzheimer’s disease (AD) and Parkinson’s disease (PD) are characterized by progressive neuronal loss, chronic microglial activation, impaired autophagic-lysosomal protein clearance, and neuroinflammation [1,7,8]. In AD, extracellular Aβ deposits and hyperphosphorylated tau neurofibrillary tangles engage microglial PRRs, driving NF-κB activation, mtROS generation, and NLRP3 inflammasome assembly [8,34]. In PD, α-synuclein aggregates and neurotoxins (such as rotenone or 6-OHDA) inhibit mitochondrial complex I, increase glycolysis, and activate substantia nigra microglia, accelerating dopaminergic degeneration [7,63,66].

GAS and HBA have been associated with improved cognitive, metabolic, and inflammatory outcomes in several neurodegeneration and vascular dementia models [8,34,39,67]. The reported measures come from brain tissue, microglial observations, and other cell types; they cannot by themselves assign the neuronal benefits to microglial AMPK/autophagy/NLRP3 signaling. For example, reductions in inflammatory markers and changes in neuronal pathology may occur in parallel without establishing which cell population initiates protection [4,34,67].

In rotenone- and 6-hydroxydopamine (6-OHDA)-induced Parkinson disease models, GAS treatment has been associated with lower microglial inflammatory readouts, dopaminergic neuron preservation, and better motor performance [7,42,66]. A rotenone-damaged PC12 cell study assessed neuronal-like glycolysis [63], whereas PINK1/Parkin-dependent mitophagy was demonstrated in a cardiac model [20]; these results should not be combined as proof of a microglial PINK1/Parkin–NLRP3 pathway in Parkinson disease. GAS-associated MEK-dependent VMAT2 trafficking provides another potential neuronal route to dopamine homeostasis [42].

Neuronal AMPK/mTOR signaling can regulate autophagy and mitochondrial maintenance, as well as processes relevant to synaptic function and survival. GAS has been tested directly in neuron-like SH-SY5Y cells under oxygen-glucose deprivation/reperfusion, where AMPK–OPA1-associated mitochondrial responses were observed [19]. In hypoxia-injured HT22 cells and vascular dementia rats, GAS instead reduced elevated LC3-II/I and Beclin-1 and increased p62 alongside PI3K/Akt activation, consistent with suppression of excessive autophagy in that model [16]. These context-dependent results caution against describing GAS as uniformly increasing neuronal autophagy; direct neuronal mTOR dependence was not established by these studies. Whether improved neuronal survival reflects a direct action on neurons, reduced microglial inflammatory signaling, or both remains unresolved. Parallel neuron-specific and inducible microglia-specific AMPK/mTOR perturbations, neuron–microglia co-cultures, and time-resolved cell-specific measurements could separate these routes of protection [16,19].

### 7.3. Neuropsychiatric Disorders

Major depressive disorder (MDD) and chronic pain–depression comorbidities involve neuroimmune dysfunction marked by microglial activation, hippocampal dendritic atrophy, and impaired bioenergetics [5,68]. Chronic psychosocial stress, systemic endotoxemia, or chronic restraint stress (CRS) triggers microglial hypertrophy, process retraction, and the release of IL-1β, TNF-α, and IL-6 in the hippocampus and medial prefrontal cortex [5,69,70]. This neuroinflammatory environment suppresses neurogenesis, reduces brain-derived neurotrophic factor (BDNF) levels, and drives behavioral despair [5,27].

GAS produces antidepressant- and anxiolytic-like effects across rodent models of LPS challenge, CRS, and diabetic encephalopathy [5,27,69]. In mice subjected to chronic LPS challenge, GAS reverses behavioral despair as evidenced by restored sucrose preference and reduced forced swim immobility by resolving hippocampal microglial hypertrophy and driving Nrf2-dependent M2-like neuroprotective polarization [5].

**Figure 6:**
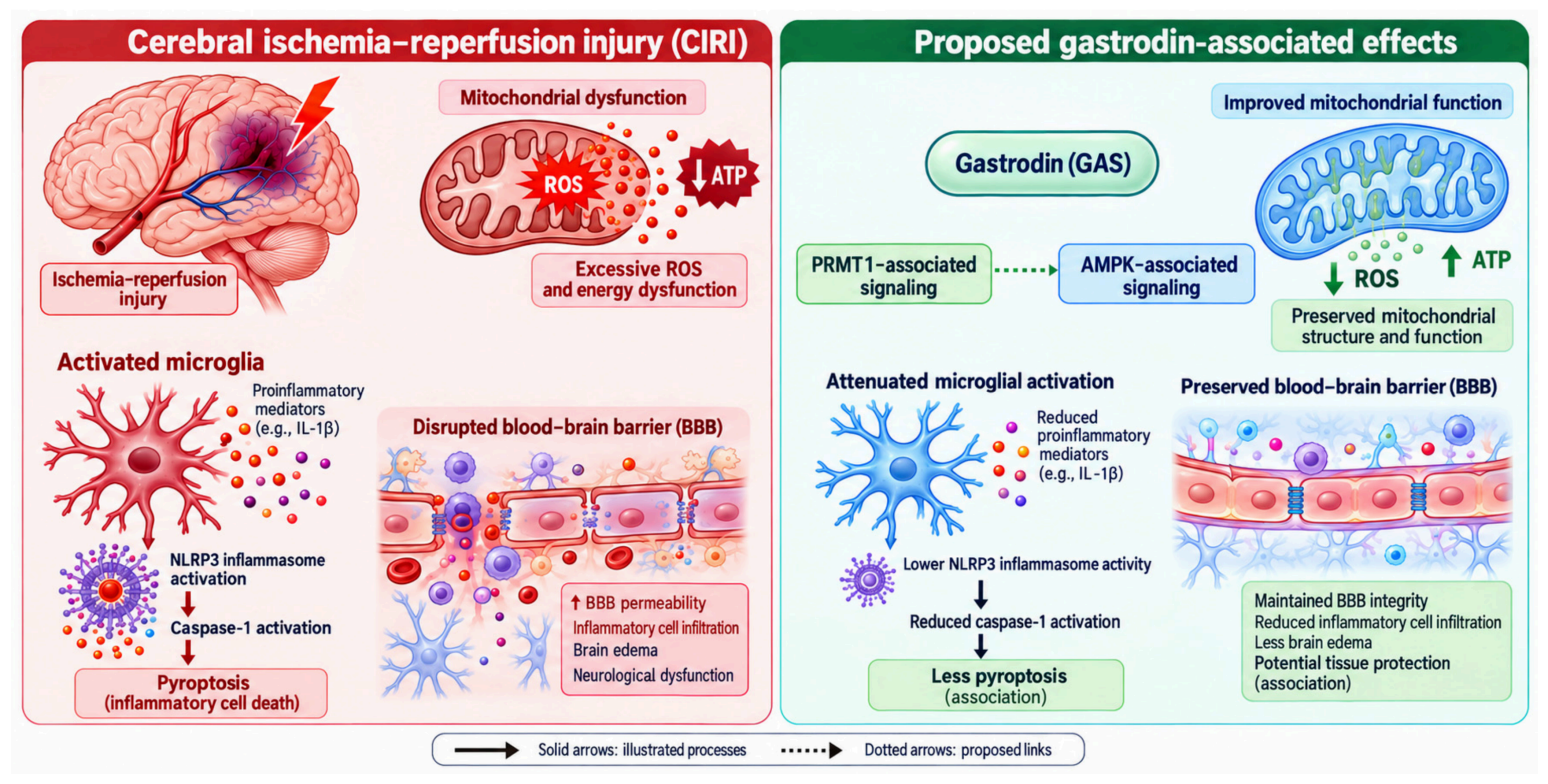
Proposed integration of PRMT1/AMPK-associated signaling, mitochondrial responses, and pyroptosis markers in cerebral ischemia–reperfusion injury (CIRI). The two panels illustrate injury-associated changes and proposed gastrodin-associated effects. Evidence for the PRMT1–AMPK association comes from endothelial and ischemia models [31], whereas mitochondrial responses [19] and pyroptosis markers [17,59] were examined in other ischemia/reperfusion models. These findings do not establish one continuous pathway in microglia. Solid arrows indicate the direction of illustrated processes; dotted arrows indicate proposed links between findings from different studies. The original schematic was prepared with Gemini (Google) assistance, and this version was redrawn with ChatGPT assistance. Abbreviations: AMPK, AMP-activated protein kinase; ATP, adenosine triphosphate; BBB, blood–brain barrier; CIRI, cerebral ischemia–reperfusion injury; GAS, gastrodin; IL-1β, interleukin-1 beta; NLRP3, NOD-like receptor family pyrin domain containing 3; PRMT1, protein arginine N-methyltransferase 1; ROS, reactive oxygen species.

In diabetic and neuropathic models displaying comorbid depression and cognitive decline, GAS suppresses endoplasmic reticulum (ER) stress, inhibits hippocampal NLRP3 activation, and reduces cleaved caspase-1 and IL-1β levels [11,27,60]. In chronic pain–depression models, GAS acts through a SIRT3-dependent pathway to deacetylate metabolic targets, suppress microglial activation, quench ROS, and restore synaptic transmission [68]. Simultaneously, GAS modulates Notch3 signaling and restores prefrontal cortical autophagic flux under stress [69,70]. Through these mechanisms, GAS targets the AMPK/autophagy/NLRP3 axis to mitigate neuroinflammation across neuropsychiatric conditions (Figure 6) [5,27,68].

## 8. Translational Gaps and Future Directions

### 8.1. The Missing Link: Identifying the Direct Molecular Target of Gastrodin

Although GAS displays anti-inflammatory, pro-autophagic, and anti-pyroptotic effects, its primary upstream molecular target remains unidentified [25,41]. Most investigations have tracked downstream endpoints such as AMPK, mTOR, and NF-κB phosphorylation, or caspase-1 and GSDMD cleavage without defining the initiating receptor or direct binding partner [10,17,33]. Natural phenolic glucosides have often been classified as non-specific antioxidants or multi-target agents, but quantitative biophysical techniques are beginning to uncover discrete, high-affinity ligand–protein interactions [8,41].

Surface plasmon resonance (SPR), CETSA, and molecular docking have identified candidate binding partners for GAS [38,41,71]. In cerebral ischemia models, GAS binds directly to PI3K (confirmed by SPR and CETSA), inducing Akt phosphorylation, AlkB homolog 5 (Alkbh5) nuclear translocation, and target mRNA demethylation [41]. In epigenetic models, GAS binds lysine acetyltransferase 8 (KAT8) and accelerates PRMT1 degradation, relieving PRMT1-mediated AMPK repression [31,71]. Molecular docking models also predict direct binding affinities between GAS derivatives and both the AMPKα catalytic domain and sirtuin-1 (SIRT1) [30]. Nevertheless, target-validation approaches including drug affinity responsive target stability (DARTS), photoaffinity labeling (PAL), and unbiased chemoproteomic profiling in primary microglia-remain underused. Future investigations should resolve co-crystal or cryo-electron microscopy (cryo-EM) structures of GAS bound to its targets to establish whether it acts as an allosteric kinase modulator, an enzyme agonist, or a membrane receptor ligand in microglia [8,25,41].

These candidate interactions should be interpreted according to the type of evidence available. Binding assays in non-microglial systems support physical interaction with particular proteins, whereas molecular docking alone predicts possible interactions and does not establish target engagement in cells [31,41,71]. It remains unknown whether any candidate is the initiating target that links GAS exposure to AMPK activation and downstream autophagy–pyroptosis regulation in microglia. Demonstrating target engagement in primary microglia, followed by target-specific loss-of-function and rescue experiments, will be needed before assigning a definitive upstream mechanism.

### 8.2. Methodological Rigor: The Need for Genetic and Pharmacological Inhibition

Pharmacological studies of natural monomers frequently rely on correlative markers rather than direct causal validation [8,25]. Confirming that GAS inhibits microglial pyroptosis via an AMPK-dependent autophagic pathway requires demonstrating that selective genetic or pharmacological deletion of key pathway nodes abolishes its therapeutic efficacy [14,33]. Chemical inhibitor experiments with Compound C, 3-MA, and Baf-A1 provide supportive pathway evidence in different models, but cannot independently establish AMPK dependence or a necessary role for autophagic flux [31,33,51,52,54,56].

Compound C affects kinases beyond AMPK, and the effect of 3-MA on autophagic flux depends on treatment conditions and duration [52,56]. Establishing causality along the AMPK/mTOR/NLRP3 axis requires genetic approaches [8]. Future studies should deploy conditional genetic models, such as microglia-specific (CX_3_CR1-CreER) AMPKα1/α2 conditional knockouts, autophagy-deficient strains (*Atg5/Atg7* knockouts), and global or conditional *Nlrp3* and *Gsdmd* knockout mice [3,19,31]. Assessing GAS efficacy in the specific absence of AMPK, Beclin-1, PINK1, or NLRP3 will determine whether GAS-induced phenotypic reprogramming depends strictly on the canonical AMPK/mTOR/NLRP3 pathway or engages compensatory networks [5,6,19]. In particular, experiments measuring autophagic flux, mitochondrial damage, inflammasome assembly, and GSDMD cleavage in the same model, with pathway-specific loss-of-function and rescue, are needed to test the proposed autophagy–pyroptosis sequence rather than infer it from separate endpoints.

The published preclinical studies use different disease models, GAS doses and routes of administration, treatment schedules, and sampling time points [5,17,31,33]. Such heterogeneity complicates comparisons of the reported autophagic, inflammasome, and functional outcomes; apparent differences should not be treated as direct contradictions unless the underlying experimental conditions are matched. Moreover, changes in LC3-II or p62 at a single time point do not, by themselves, establish autophagic flux [55]. Future studies should report these experimental variables consistently and assess autophagic flux and pyroptosis at multiple time points in the same model. Representative protocols and outcome measures are compared in Supplementary Table S1. The experimental systems, GAS regimens, molecular readouts, and relevance to microglial autophagy–pyroptosis crosstalk are summarized in Supplementary Table S2.

The reliance on pharmacological inhibition also limits causal inference. Compound C inhibits kinases beyond AMPK [52], whereas 3-MA can either suppress or promote autophagic flux depending on the treatment conditions and duration [56]. Thus, inhibitor-based reversal of a GAS effect should be interpreted alongside genetic loss-of-function and rescue experiments. Inducible, microglia-specific deletion of AMPKα or *Atg5/Atg7* could test whether these nodes are required for GAS-mediated suppression of NLRP3 activation and GSDMD cleavage; complementary *Nlrp3* or *Gsdmd* deletion could clarify the contribution of pyroptosis to tissue protection. Parallel measurement of autophagic flux, mitochondrial damage, inflammasome assembly, and functional outcomes would strengthen the proposed causal sequence [55].

### 8.3. Enhancing CNS Delivery for Clinical Translation

Clinical translation of GAS is constrained by uncertain human CNS exposure and a limited duration of systematic safety follow-up [8,24,72]. Oral bioavailability varies across experimental species and strains, and rat brain-to-plasma exposure cannot be assumed to predict human microglial target engagement (Table 1). Short-term clinical use and a seven-day randomized trial provide initial tolerability information, but neither establishes a safe chronic dose or a therapeutic window for neurodegenerative and neuropsychiatric conditions [72].

Human studies illustrate both the clinical interest in GAS and the remaining mechanistic gap. In a randomized, double-blind, placebo-controlled cardiac surgery trial, GAS infusion was associated with a lower incidence of postoperative delirium than placebo, but the study did not measure microglial autophagy, NLRP3 activation, or pyroptosis [72]. A published protocol describes the EASTERN multicenter placebo-controlled trial of oral GAS for medication-overuse headache; as a protocol, it provides no efficacy results [73]. These disease-specific studies cannot establish that the AMPK/mTOR/NLRP3 pathway mediates clinical benefit in neurodegenerative or neuroinflammatory disorders.

Most mechanistic conclusions concerning the AMPK/mTOR/NLRP3 axis derive from cellular and animal experiments [5,17,31,33]. Human studies have not yet directly linked GAS exposure to microglial target engagement, autophagy–pyroptosis regulation, and neurological outcomes. Accordingly, this mechanistic chain remains a preclinical hypothesis rather than an established explanation of clinical benefit.

GAS-loaded delivery systems have shown promising results outside the CNS: a responsive hydrogel carrying targeted nanoparticles was studied in cardiac fibrosis [21], and a biomimetic nanosystem was studied in acute lung injury [74]. Neither study establishes brain accumulation or microglial uptake. Brain-directed carriers and focused ultrasound-mediated BBB opening are potential delivery strategies, but their GAS-specific CNS exposure, cell selectivity, efficacy, and safety require direct testing [8]. These measurements, together with well-controlled clinical studies, are needed before GAS can be described as a validated microglial-directed therapy [25,75].

### 8.4. Safety, Toxicology, and Long-Term Use

A translational assessment must distinguish purified GAS, its aglycone HBA, and whole-plant preparations. A 28-day oral study of a water extract of *Gastrodia elata* reported no significant genotoxic signal in the assays used and a no-observed-adverse-effect level (NOAEL) of at least 8065 mg/kg/day in mice [76]. This is a preparation-specific finding and cannot be assigned to purified GAS or HBA. The reviewed evidence does not establish validated compound-specific oral or parenteral LD50 values, repeat-dose NOAELs, exposure-based safety margins, or a therapeutic window for GAS or HBA. Acute tolerability at an experimental dose cannot substitute for chronic toxicity testing.

For both compounds, the available studies do not comprehensively characterize dose- and duration-dependent injury to liver, kidney, heart, or CNS; genotoxicity; reproductive and developmental toxicity; or carcinogenic risk. HBA is an active, systemically detectable metabolite, but its toxicology and brain exposure cannot be inferred from the parent compound or the plant extract [48,49]. Direct, exposure-matched GAS–HBA comparisons are needed, including repeated dosing, clinical chemistry, histopathology, neurobehavioral assessments, and developmental endpoints. Quantitative inhibition and induction studies for major cytochrome P450 enzymes, relevant transporters, and clinically co-administered medicines are also needed before predicting drug–drug interaction risk; docking or metabolic plausibility alone does not establish a clinical interaction.

Human data provide limited short-term reassurance rather than a defined long-term safe dose. In a placebo-controlled cardiac-surgery trial, 600 mg GAS was infused twice daily for seven days; adverse events occurred in 7/77 GAS-treated participants and 11/78 placebo-treated participants, with none judged drug-related [72]. A retrospective series of 315 reported GAS-associated adverse drug reaction/event cases described mainly gastrointestinal and skin manifestations, but without an exposed population denominator it cannot estimate incidence [77]. The seven-day intravenous regimen is an observed trial exposure, not a recommended chronic dose range, and comparable clinical safety data for isolated HBA are lacking. Longitudinal trials should prospectively monitor laboratory indices, organ function, neurobehavior, adverse events, and concomitant medication use over the duration intended for chronic CNS disease.

## 9. Conclusions

Microglial activation and neuroinflammation occur in many central nervous system disorders. GAS has shown anti-inflammatory and neuroprotective effects in preclinical studies, including experiments that assess microglial responses [3,4,5]. The AMPK/mTOR/NLRP3 framework assembled in this review links potentially relevant processes, but its complete sequence has not been verified as a GAS-dependent mechanism in microglia.

Evidence for individual components comes from different experimental systems. PRMT1–AMPK signaling was examined in brain microvascular endothelial cells [31], AMPK–OPA1 mitochondrial remodeling in ischemia/reperfusion models [19], and autophagy regulation in other cell types [15,33]. GAS and HBA altered brain energy-metabolism measures in a vascular dementia model [39]. These observations cannot establish that GAS directly initiates the same AMPK/mTOR–mitophagy sequence in microglia.

Preclinical studies separately report changes in inflammasome or pyroptosis markers after GAS treatment in traumatic brain injury, ischemia, and microvascular injury [3,10,17]. Mitophagy and mitochondrial damage provide a plausible biological link to NLRP3 activation, but whether GAS-dependent autophagic flux is required to suppress microglial NLRP3 assembly, caspase-1 activation, and GSDMD cleavage remains untested in a matched causal design [19,55].

GAS promotes an Arg-1-positive microglial phenotype in an LPS-challenged mouse model [5]. Glycolytic and mitochondrial outcomes, however, have often been measured in whole brain tissue or non-microglial cells [39,63]. The proposed coupling of reduced pyroptosis, restored oxidative metabolism, and microglial phenotypic recovery is therefore a testable interpretation rather than a demonstrated sequence; M1/M2 markers alone do not capture the full range of microglial states.

**Figure 7:**
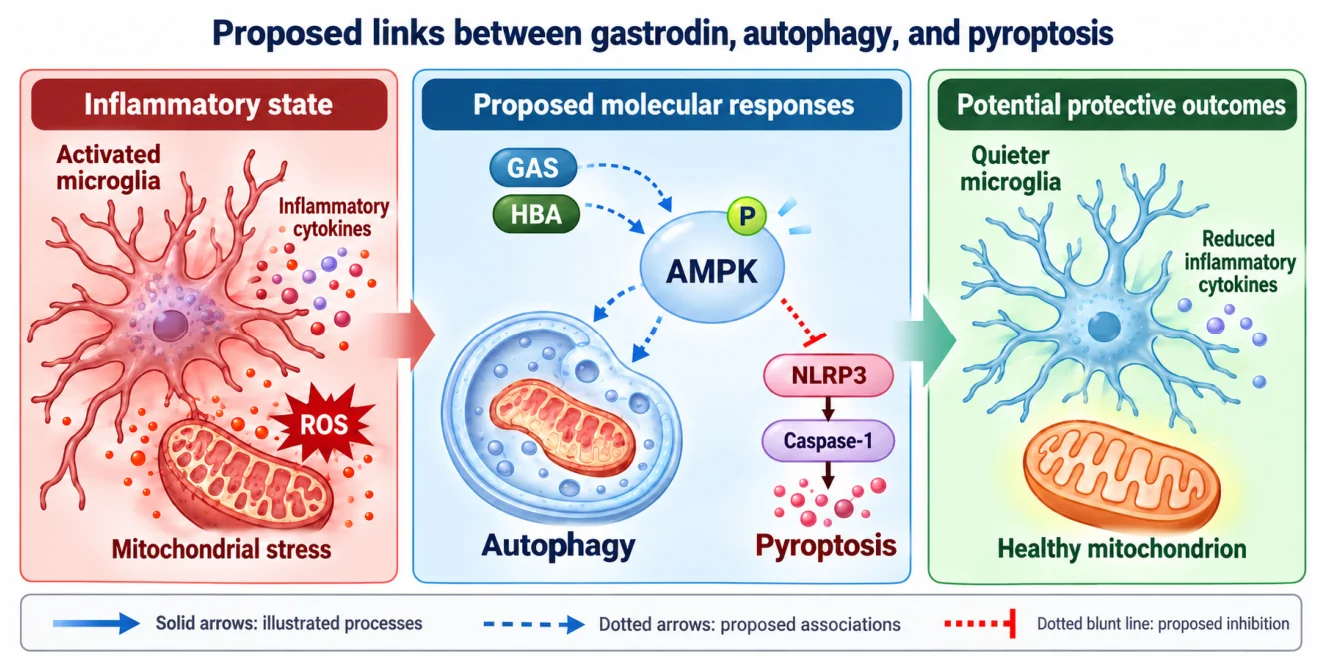
Proposed links between gastrodin, autophagy, and pyroptosis. This schematic integrates findings from microglial phenotype studies [5], mitochondrial and AMPK studies [19,31], and inflammasome/pyroptosis studies [3,17]. The illustrated AMPK–autophagy–NLRP3 relationships are proposed associations and have not been established as one continuous causal pathway in microglia. Solid arrows indicate illustrated processes, dotted arrows indicate proposed associations, and the dotted blunt-ended line indicates proposed inhibition. The original schematic was prepared with Gemini (Google) assistance; this version was redrawn with ChatGPT assistance. Abbreviations: AMPK, AMP-activated protein kinase; GAS, gastrodin; HBA, p-hydroxybenzyl alcohol; NLRP3, NOD-like receptor family pyrin domain containing 3; ROS, reactive oxygen species.

The next priority is to test the proposed sequence in the same microglial system using time-resolved autophagic flux, mitochondrial damage, NLRP3 assembly, and GSDMD cleavage assays. Inducible microglia-specific AMPKα and *Atg5/Atg7* deletion with rescue, together with *Nlrp3* or *Gsdmd* gene perturbation, could clarify necessity and order of events. Matched GAS/HBA exposure and target-engagement studies would help assign effects to the parent compound or metabolite. Delivery strategies and human trials warrant further study, but current clinical data do not establish an AMPK/mTOR/NLRP3 mechanism in patients. Figure 7 therefore depicts a hypothesis to be tested, not a confirmed microglial signaling cascade. Neuron-specific AMPK or mTOR perturbation should be compared with microglia-specific perturbation in the same model to test whether neuronal rescue is cell autonomous or mediated through altered microglial signaling.

## Data Availability

No new data were created or analyzed in this study. Data sharing is not applicable to this article.

## Supplementary Materials

The following supporting information can be downloaded at: https://www.mdpi.com/article/10.3390/ijms27198754/s1.

## Abbreviations

The following abbreviations are used in this manuscript:

2-VO	Two-vessel occlusion
3-MA	3-Methyladenine
4EBP1	Eukaryotic translation initiation factor 4E-binding protein 1
ACC	Acetyl-CoA carboxylase
AD	Alzheimer’s disease
AMPK	AMP-activated protein kinase
ASC	Apoptosis-associated speck-like protein containing a CARD
ATP	Adenosine triphosphate
BBB	Blood–brain barrier
BCL6	B-cell lymphoma 6
CETSA	Cellular thermal shift assay
CIRI	Cerebral ischemia–reperfusion injury
CNS	Central nervous system
COX-2	Cyclooxygenase-2
DAMPs	Damage-associated molecular patterns
ER	Endoplasmic reticulum
FoxO1	Forkhead box O1
GAS	Gastrodin
GLUTs	Glucose transporters
GSDMD	Gasdermin D
GSDMD-N	N-terminal fragment of gasdermin D
HBA	*p*-Hydroxybenzyl alcohol
HK2	Hexokinase 2
IKK	IκB kinase
IL	Interleukin
iNOS	Inducible nitric oxide synthase
LDH	Lactate dehydrogenase
LDHA	Lactate dehydrogenase A
LPS	Lipopolysaccharide
MAPK	Mitogen-activated protein kinase
MCAO/R	Middle cerebral artery occlusion and reperfusion
mTOR	Mechanistic target of rapamycin
mTORC1	Mechanistic target of rapamycin complex 1
mtROS	Mitochondrial reactive oxygen species
NF-κB	Nuclear factor-κB
NLRP3	NOD-like receptor family pyrin domain-containing 3
Nrf2	Nuclear factor erythroid 2-related factor 2
OGD/R	Oxygen–glucose deprivation/reperfusion
OMM	Outer mitochondrial membrane
OPA1	Optic atrophy 1
OPTN	Optineurin
ox-mtDNA	Oxidized mitochondrial DNA
OXPHOS	Oxidative phosphorylation
PD	Parkinson’s disease
PI3K	Phosphoinositide 3-kinase
PINK1	PTEN-induced kinase 1
PKM2	Pyruvate kinase M2
PPP	Pentose phosphate pathway
PRMT1	Protein arginine N-methyltransferase 1
ROS	Reactive oxygen species
SGLT1	Sodium-dependent glucose transporter 1
SIRT3	Sirtuin 3
SPR	Surface plasmon resonance
SQSTM1/p62	Sequestosome 1
STAT3	Signal transducer and activator of transcription 3
TBI	Traumatic brain injury
TCA	Tricarboxylic acid
TFAM	Mitochondrial transcription factor A
TFEB	Transcription factor EB
TLR4	Toll-like receptor 4
TNF-α	Tumor necrosis factor-α
TSC2	Tuberous sclerosis complex 2
ULK1	Unc-51-like autophagy-activating kinase 1
VMAT2	Vesicular monoamine transporter 2
