# Supplementary material for: Gastrodin and Microglial Autophagy–Pyroptosis Crosstalk: Evidence and Hypotheses Involving the AMPK/mTOR/NLRP3 Axis
Source: Int J Mol Sci. 2026 Sep 30;27(19):8754. doi: 10.3390/ijms27198754 (PMC13665310; doi:10.3390/ijms27198754)
Supplement: Supplementary file 1 [file ijms-27-08754-s001.zip › ijms-4597668-supplementary.pdf]

Supplementary Table S1. Representative preclinical studies of gastrodin in neurological models

Studies were selected to illustrate variation in model and treatment regimen. The table is descriptive and does not imply that the proposed AMPK–autophagy–NLRP3 sequence was tested as a whole in any one study.

Study	Preclinical model	Gastrodin regimen	Major readouts	Main finding and scope
Yang et al. [3]	Weight-drop traumatic brain injury; male rats	15, 30, or 60 mg/kg, intraperitoneally, once daily for 7 days after injury	Neurological score; histology; brain NLRP3, ASC, caspase-1/11, GSDMD; inflammatory cytokines	Less tissue injury and lower inflammatory and pyroptosis-associated markers. Whole brain readouts do not establish microglia-specific effects.
Zhang et al. [5]	Repeated LPS challenge; male C57BL/6 mice (LPS 0.25 mg/kg/day for 10 days)	25, 50, or 100 mg/kg/day; 13-day gastrodin intervention	Hippocampal microglial morphology and Arg-1; Nrf2; inflammatory mediators; behavioral tests	Shift toward Arg-1-positive microglia and improved depression/anxiety-like behavior; Nrf2 inhibitor opposed the response.
Wu et al. [39]	Two-vessel occlusion; rat vascular dementia model	25 or 50 mg/kg, oral gavage, daily for 4 weeks; HBA comparator at 25 or 50 mg/kg	Spatial learning/memory; hippocampal pathology; brain metabolomics and mitochondrial function	Gastrodin and HBA improved cognitive and metabolic readouts. The study does not isolate a microglial AMPK–NLRP3 mechanism.
Yin et al. [67]	APP/PS1 transgenic mouse model of Alzheimer disease	10 or 20 mg/kg, intragastrically, once daily; mechanism arm used 20 mg/kg	Morris water maze and eight-arm maze; inflammatory cytokines; PPARγ and NF-κB signaling	Improved cognitive and inflammatory readouts; PPARγ silencing attenuated the reported gastrodin effect.

Abbreviations: AMPK, AMP-activated protein kinase; Arg-1, arginase 1; ASC, apoptosis-associated speck-like protein containing a CARD; GSDMD, gasdermin D; HBA, p-hydroxybenzyl alcohol; LPS, lipopolysaccharide; NF-κB, nuclear factor kappa B; NLRP3, NOD-like receptor family pyrin domain containing 3; Nrf2, nuclear factor erythroid 2-related factor 2; PPARγ, peroxisome proliferator-activated receptor gamma.

Supplementary Table S2. Experimental evidence relevant to gastrodin and microglial autophagy–pyroptosis crosstalk

The table distinguishes findings obtained in microglia from those reported in other cells or whole tissues. Pathway components measured in different experimental systems do not establish one causal mechanism in microglia. NR denotes a regimen detail that could not be verified from the accessible primary report; “not assessed” means that the indicated endpoint was not reported in this summary.

Study	Disease / model	Experimental system	GAS dose / route	Molecular pathway	Autophagy / pyroptosis endpoints	Major finding and evidential scope
[2] Yao et al. 2019	LPS activation	BV-2 microglial cells (direct microglial cell-line evidence)	10–80 μM; culture exposure	Wnt/β-catenin	Not assessed	Reduced proliferation and inflammatory responses; no AMPK/mTOR/NLRP3 test.
[3] Yang et al. 2022	Weight-drop traumatic brain injury	Rat brain homogenate (mixed cells; microglia not isolated)	15, 30, 60 mg/kg; intraperitoneal, daily for 7 days	NLRP3/ASC/caspase-1	GSDMD, caspase-1/11, NLRP3, ASC, IL-1β, IL-18; autophagy not assessed	Less injury and lower pyroptosis-associated markers; cellular origin unresolved.
[5] Zhang et al. 2023	Repeated LPS challenge and depressive/anxiety-like behavior	Mouse hippocampal microglia in vivo (microglia-associated measurements)	25, 50, 100 mg/kg/day; route NR	Nrf2-dependent phenotype regulation	Arg-1 and morphology; autophagy/pyroptosis not assessed	More Arg-1-positive microglia and better behavioral readouts; does not test AMPK/NLRP3.
[9] Dai et al. 2011	LPS activation	BV-2 microglial cells (direct microglial cell-line evidence)	30, 40, 60 μM; culture pretreatment	ERK/JNK/p38 MAPK	Not assessed	Reduced iNOS, COX-2, and inflammatory cytokines; no autophagy/pyroptosis test.
[10] Sun et al. 2019	Cardiac microvascular reperfusion injury	Cardiac microvascular endothelial cells and cardiac tissue (non-CNS)	Dose / route NR	NLRP3/caspase-1	Pyroptosis and IL-1β; autophagy not assessed	Lower endothelial pyroptosis; supports biological plausibility only for microglia.
[17] Zhang et al. 2021	Cerebral ischemia/reperfusion	MCAO rat brain and OGD/R neuronal cells (not isolated microglia)	Dose NR; intraperitoneal in rats, 7 days before and after injury	NEAT1/miR-22-3p/NLRP3	NLRP3, cleaved caspase-1, IL-1β, IL-18; autophagy not assessed	Reduced infarct and pyroptosis-associated markers; neuronal and mixed-tissue evidence.
[19] Liu et al. 2025	Cerebral ischemia/reperfusion	MCAO/R rats and OGD/R SH-SY5Y cells (not microglia)	Dose / route NR	AMPK/OPA1 mitochondrial fusion	Mitochondrial morphology/function; autophagic flux and pyroptosis not assessed	Mitochondrial protection supports a distinct upstream branch, not the full microglial axis.
[31] Luo et al. 2026	Cerebral ischemia/reperfusion	tMCAO mice and OGD/R human brain microvascular endothelial cells	Dose / route NR	PRMT1/AMPK	Ferroptosis and oxidative stress; autophagy/pyroptosis not assessed	AMPK-linked endothelial protection; does not establish microglial pathway dependence.
[39] Wu et al. 2023	Two-vessel occlusion / vascular dementia	Rat brain tissue (mixed cells); HBA comparator	25 or 50 mg/kg, oral gavage for 4 weeks	Energy metabolism and mitochondrial function	Autophagy/pyroptosis not assessed	Improved cognitive and metabolic readouts; not specific to microglia.
[67] Yin et al. 2024	APP/PS1 Alzheimer model	Transgenic mouse brain and amyloid-stimulated microglial cell assays	10 or 20 mg/kg, intragastric daily in mice	PPARγ/NF-κB	Inflammatory cytokines; autophagy/pyroptosis not assessed	Improved cognition and lower neuroinflammation; a separate signaling branch.

Abbreviations: AMPK, AMP-activated protein kinase; ASC, apoptosis-associated speck-like protein containing a CARD; COX-2, cyclooxygenase-2; GAS, gastrodin; GSDMD, gasdermin D; HBA, p-hydroxybenzyl alcohol; iNOS, inducible nitric oxide synthase; LPS, lipopolysaccharide; MCAO/R, middle cerebral artery occlusion/reperfusion; NEAT1, nuclear paraspeckle assembly transcript 1; NLRP3, NOD-like receptor family pyrin domain containing 3; NR, not reported here; OGD/R, oxygen-glucose deprivation/reoxygenation; OPA1, optic atrophy 1; PRMT1, protein arginine N-methyltransferase 1; tMCAO, transient middle cerebral artery occlusion. Reference numbers correspond to the main manuscript.
